# Association between circulating tumor necrosis factor-related biomarkers and estimated glomerular filtration rate in type 2 diabetes

**DOI:** 10.1038/s41598-018-33590-w

**Published:** 2018-10-17

**Authors:** Nozomu Kamei, Mami Yamashita, Yuji Nishizaki, Naotake Yanagisawa, Shuko Nojiri, Kanako Tanaka, Yoshinori Yamashita, Terumi Shibata, Maki Murakoshi, Yusuke Suzuki, Tomohito Gohda

**Affiliations:** 1 Department of Endocrinology and Metabolism, Hiroshima Red Cross Hospital and Atomic-bomb Survivors Hospital, 1-9-6, Senda-machi, Naka-ku, Hiroshima, 730-8619 Japan; 2 Department of Endocrinology and Diabetology, National Hospital Organization, Kure Medical Center and Chugoku Cancer Center, 3-1, Aoyama-cho, Kure-city, Hiroshima, 737-0023 Japan; 3 Institute for Clinical Research, National Hospital Organization, Kure Medical Center and Chugoku Cancer Center, 3-1, Aoyama-cho, Kure-city, Hiroshima, 737-0023 Japan; 4 Department of Molecular and Internal Medicine, Graduate School of Biomedical and Health Sciences, Hiroshima University, 1-2-3, Kasumi, Minami-ku, Hiroshima, 734-8551 Japan; 5 Juntendo University, Medical Technology Innovation Center, 2-1-1, Hongo, Bunkyo-ku, Tokyo, 113-8421 Japan; 6 Department of Nephrology, Juntendo University Faculty of Medicine, 2-1-1, Hongo, Bunkyo-ku, Tokyo, 113-8421 Japan

## Abstract

Chronic inflammation plays a crucial role in the development/progression of diabetic kidney disease. The involvement of tumor necrosis factor (TNF)-related biomarkers [TNFα, progranulin (PGRN), TNF receptors (TNFR1 and TNFR2)] and uric acid (UA) in renal function decline was investigated in patients with type 2 diabetes (T2D). Serum TNF-related biomarkers and UA levels were measured in 594 Japanese patients with T2D and an eGFR ≥30 mL/min/1.73 m^2^. Four TNF-related biomarkers and UA were negatively associated with estimated glomerular filtration rate (eGFR). In a logistic multivariate model, each TNF-related biomarker and UA was associated with lower eGFR (eGFR <60mL /min/1.73 m^2^) after adjustment for relevant covariates (basic model). Furthermore, UA and TNF-related biomarkers other than PGRN added a significant benefit for the risk factors of lower eGFR when measured together with a basic model (UA, ΔAUC, 0.049, p < 0.001; TNFα, ΔAUC, 0.022, p = 0.007; TNFR1, ΔAUC, 0.064, p < 0.001; TNFR2, ΔAUC, 0.052, p < 0.001) in receiver operating characteristic curve analysis. TNFR ligands were associated with lower eGFR, but the associations were not as strong as those with TNFRs or UA in patients with T2D and an eGFR ≥30 mL/min/1.73 m^2^.

## Introduction

Chronic low-grade inflammation may induce chronic kidney disease (CKD) in patients with diabetes. Tumor necrosis factor alpha (TNFα) is a central pro-inflammatory cytokine produced by both macrophages and innate kidney cells such as podocytes, mesangial cells, epithelial cells, and endothelial cells^1^. Treatment with pentoxifylline, a drug that can inhibit TNFα production, significantly reduced albuminuria and slowed estimated glomerular filtration ratio (eGFR) decline in patients with type 2 diabetes (T2D) and stage 3–4 CKD^2^. Awad *et al*.^3^ demonstrated that macrophage-derived TNFα plays a particularly important role in diabetic renal injury using macrophage-specific TNFα-deficient mice. Circulating levels of TNF receptors (TNFRs; TNFR1, TNFR2), which are the surface receptors of TNFα, in patients with diabetes have been associated with renal traits (albuminuria and eGFR) as well as cardiovascular disease (CVD) and all-cause mortality in both cross-sectional and longitudinal studies^4–16^.

Progranulin (PGRN), a recently recognized adipokine related to obesity and diabetes, also binds to TNFRs^17,18^. PGRN appeared to have protective effects against endotoxin-induced acute kidney injury or renal ischemia/reperfusion injury in mice models^19,20^. Several studies have shown that serum levels of PGRN were elevated in patients with diabetes and macroalbuminuria (Macro) or reduced renal function^21–23^. However, little is known about whether PGRN is associated with classical TNF-related biomarkers (TNFα and TNFRs).

Uric acid (UA) has recently re-entered the spotlight because hyperuricemia is not only a result of reduced renal clearance or dysfunctional handling by proximal tubules but also a cause of kidney injury^24,25^. Kim *et al*.^26^ demonstrated that hyperuricemia-induced Nod-like receptor protein 3 (NLRP3) activation of macrophages contributes to the progression of renal injury in a rat model of T2D. Moreover, treatment with Febuxostat, a non-purine xanthine oxidase-specific inhibitor, improved renal expression of inflammatory molecules such as E-selectin and vascular cell adhesion molecule 1 (VCAM-1) and prevented macrophage infiltration in the kidneys of streptozotocin (STZ)-induced diabetic rats^27^.

However, careful consideration of the relationships among these biomarkers has not yet been performed. Therefore, the aim of this study was to determine the associations among TNF-related biomarkers, UA, and renal function in a large cohort of Japanese patients with T2D and eGFR ≥30 mL/min/1.73 m^2^.

## Material and Methods

### Study design

Japanese patients with diabetes were recruited for observation of the natural course of diabetic kidney disease (DKD) at Kure Medical Center and Chugoku Cancer Center between July 1, 2014 and March 31, 2016. Patients with type 1 diabetes or secondary diabetes, and those with eGFR <30 mL/min/1.73 m^2^ (stage 4–5 CKD) were excluded from this study. A total of 594 patients with T2D and available baseline serum data were ultimately included. This study was approved by the ethics committee of Kure Medical Center and Chugoku Cancer Center. Informed consent was obtained from all patients, and the study complied with the guidelines of the Declaration of Helsinki.

Each patient’s baseline anthropometric and clinical characteristics were recorded. Blood pressure (BP) was measured using an automated sphygmomanometer (HBP-9020; Omron healthcare Co., Ltd., Kyoto, Japan) with patients in the sitting position after a rest period of at least 5 minutes. Blood and spot urine specimens were obtained for laboratory analyses. Body mass index was calculated as weight/height^2^ (kg/m^2^). Serum creatinine (Cr) was measured in a central laboratory (SRL Co., Ltd., Hachioji, Japan) by means of the enzymatic Cr assay method using the liquid Cr measurement reagent Determiner L CRE (Kyowa Medex Co., Ltd., Tokyo, Japan) and a BioMajesty JCA-BM8060 auto-analyzer (JEOL, Tokyo, Japan) to calculate eGFR. To develop an eGFR equation for the Japanese population by modifying the isotope dilution mass spectrometry (IDMS)-traceable Modified Diet in Renal Disease (MDRD) equation, serum Cr and inulin clearance were simultaneously measured under an initiative of the Japanese Society of Nephrology (JSN) in the past: eGFR (mL/min/1.73 m^2^) = 194 × [age (years)]^−0.287^ × [serum Cr (mg/dL)]^−1.904^ × 0.739 (for females)^28^. Baseline serum samples were obtained and stored at −80 °C until use.

### Laboratory measurements

We used enzyme-linked immunosorbent assay to measure TNFR1, TNFR2, PGRN (cat. nos. DRT100, DRT200, DPGRN0; R&D Systems, Minneapolis, MN, USA) and total TNFα (cat. no. KAC1751; Invitrogen, Carlsbad, CA, USA) as described previously^11,29^. Two internal serum controls were included in each assay to estimate inter-assay coefficient of variation. The inter-assay coefficient of variation for TNF-related biomarkers was consistently <10% (TNFα, 7.0%; PGRN, 6.8%; TNFR1, 8.0%; TNFR2, 9.4%). C-reactive protein (CRP), UA, lipids, hemoglobin, and hemoglobin A1c (HbA1c) were measured at Kure Medical Center and Chugoku Cancer Center using routine laboratory methods. Non-high-density lipoprotein cholesterol (non-HDL-C) levels were defined as the difference between total cholesterol and HDL-C levels. Urinary albumin and Cr were quantified using immunonephelometry (N-assay TIA Micro Alb; Nittobo Medical Co., Ltd., Fukushima, Japan) and an enzymatic method respectively. Urinary albumin to creatinine ratio (ACR) was expressed as milligrams per gram of Cr.

### Statistical analyses

All variables were expressed as percentage values for categorical data and represented as mean (SD) or median (interquartile range) for continuous data. The distribution of concentration of biomarkers was right-skewed, and four TNF-related biomarkers, UA, CRP, ACR, and eGFR were handled as continuous variables after common logarithmic transformation. For analytical purposes, patients were stratified based on their eGFR [eGFR <60 vs. ≥60 (mL/min/1.73 m^2^)] or ACR [ACR <30, normoalbuminuria (Normo); ACR 30–299, microalbuminuria (Micro); ACR ≥300, Macro (mg/g·Cr)] levels. Differences between groups were analyzed using Student’s *t*-test for continuous variables and chi-square test for dichotomous variables, or one-way analysis of variance when comparing more than two groups. Pearson correlation analysis was used to assess associations among renal traits (ACR and eGFR), UA, and TNF-related biomarkers.

Univariate logistic regression analyses were used to examine the factors associated with lower eGFR. Next, using a multivariate logistic regression model, we evaluated the association of UA or TNF-related biomarkers with lower eGFR. Candidate covariates were selected as follows: First, age and sex were included into adjusted models based on biological plausibility; next, traditional confounders of DKD such as systolic BP (Sys BP), HbA1c, and ACR were also included in the adjusted models based on findings from prior studies. Finally, the following covariate associated with eGFR was considered for inclusion based on findings from univariate logistic regression analysis in the present study: hemoglobin (OR, 0.70; 95% CI, 0.63–0.78; p < 0.001). Bonferroni correction was applied for selection of covariates (n = 11), i.e., p-value < 0.0045. The basic model eventually consisted of six covariates (age, sex, hemoglobin, Sys BP, HbA1c, and ACR). To examine the additive benefit of TNF-related biomarkers and UA in comparison with the basic model alone as risk factors of increased ACR, we added eGFR instead of ACR for selection of covariates. Each biomarker was added individually and then in pairwise combinations. Because TNFR1 and TNFR2 were very strongly correlated, they were not included in the model simultaneously. The contribution of each TNF-related biomarker or UA as a risk factor of lower eGFR (eGFR < 60 mL/min/1.73 m^2^) and increased ACR (ACR ≥ 30) was calculated based on area under the receiver operating characteristic (ROC) curve (AUC). The optimal cut-off points of each TNF-related biomarker and UA for risk of lower eGFR was obtained from the point on the ROC curve closest to (0, 1). Statistical analysis was performed using the software SAS 9.4 (SAS Institute, Cary, NC, USA). A two-sided p-value < 0.05 was considered statistically significant.

## Results

### Baseline patient characteristics

The mean (±SD) age of the study population was 65 (±13) years; 329 (55.4%) patients were men and 260 (43.8%) patients had increased ACR (Micro or Macro). Median (25^th^–75^th^ percentile) eGFR was 69 (56–84) mL/min/1.73 m^2^.

Clinical characteristics of the 594 patients with diabetes stratified based on eGFR (mL/min/1.73 m^2^) (≥60,<60) or ACR (mg/g·Cr) [<30 (Normo), 30–299 (Micro), ≥300 (Macro)] are summarized in Table 1 and Supplementary Table 1. Patients in the eGFR <60 group were older and tended to be male; had higher frequencies of increased ACR; had a higher UA level and ACR; and had lower HDL-C, hemoglobin, and HbA1c levels. In addition, serum levels of TNFα, PGRN, TNFR1, and TNFR2 in the eGFR <60 group patients were significantly higher than those in the eGFR ≥60 group patients, although CRP levels did not differ between the groups.

**Table 1:** Characteristics of the study group by eGFR level.

Characteristic	All	eGFR ≥ 60	eGFR 30–59	*P* ^ a^
	(N = 594)	(n = 402)	(n = 192)	
eGFR (mL/min/1.73 m^2^)	69 (56, 84)	80 (69, 92)	48 (43, 55)	
ACR (mg/g·Cr)	22 (9, 123)	18.5 (8.0, 58.3)	66 (13, 360)	<0.001
Normo (%)	56.2	63.7	40.6	<0.001
Micro (%)	28.8	27.6	31.3	
Macro (%)	15.0	8.7	28.1	
Age (yr)	65 ± 13	62 ± 13	71 ± 10	<0.001
Male sex (%)	55.4	52.0	62.5	0.01
BMI (kg/m^2^)	25.1 ± 4.6	25.3 ± 4.9	24.7 ± 3.9	0.13
Sys BP (mmHg)	139 ± 17	138 ± 16	141 ± 20	0.13
UA (mg/dL)	5.4 ± 1.3	5.0 ± 1.3	6.0 ± 1.3	<0.001
HDL-C (mg/dL)	52 ± 13	53 ± 13	50 ± 13	0.007
Non-HDL-C (mg/dL)	130 ± 32	131 ± 33	127 ± 32	0.12
Hemoglobin (g/dL)	13.6 ± 1.7	13.9 ± 1.6	12.9 ± 1.7	<0.001
HbA1c (%)	7.3 ± 1.2	7.4 ± 1.2	7.2 ± 1.1	0.03
CRP (mg/dL)	0.11 (0.06, 0.19)	0.10 (0.06, 0.19)	0.11 (0.07, 0.19)	0.28
**TNF-related biomarkers**				
TNFα (pg/mL)	12.6 (9.5, 18.3)	11.1 (8.8, 15.8)	15.9 (12.2, 21.5)	<0.001
PGRN (ng/mL)	56 (49, 65)	55 (47, 63)	60 (52, 67)	<0.001
TNFR1 (pg/mL)	1562 (1263, 2016)	1384 (1169, 1691)	2149 (1695, 2627)	<0.001
TNFR2 (pg/mL)	3339 (2717, 4297)	2972 (2535, 3610)	4337 (3696, 5635)	<0.001

^a^eGFR ≥60 vs. eGFR 30–59. Data are mean ± SD, median (quartiles), or %.ACR, the ratio of urinary albumin to creatinine; BMI, body mass index; CRP, C-reactive protein; eGFR, estimated glomerular filtration rate; HbA1c, hemoglobin A1c; HDL-C, high-density lipoprotein cholesterol; Micro, microalbuminuria; Normo, noromoalbuminuria; PGRN, progranulin; Macro, macroalbuminuria; SD, standard deviation; Sys BP, systolic blood pressure; TNFR, TNF receptor; UA, uric acid.

### Correlation among TNF-related biomarkers, UA, eGFR and ACR

**Figure 1:**
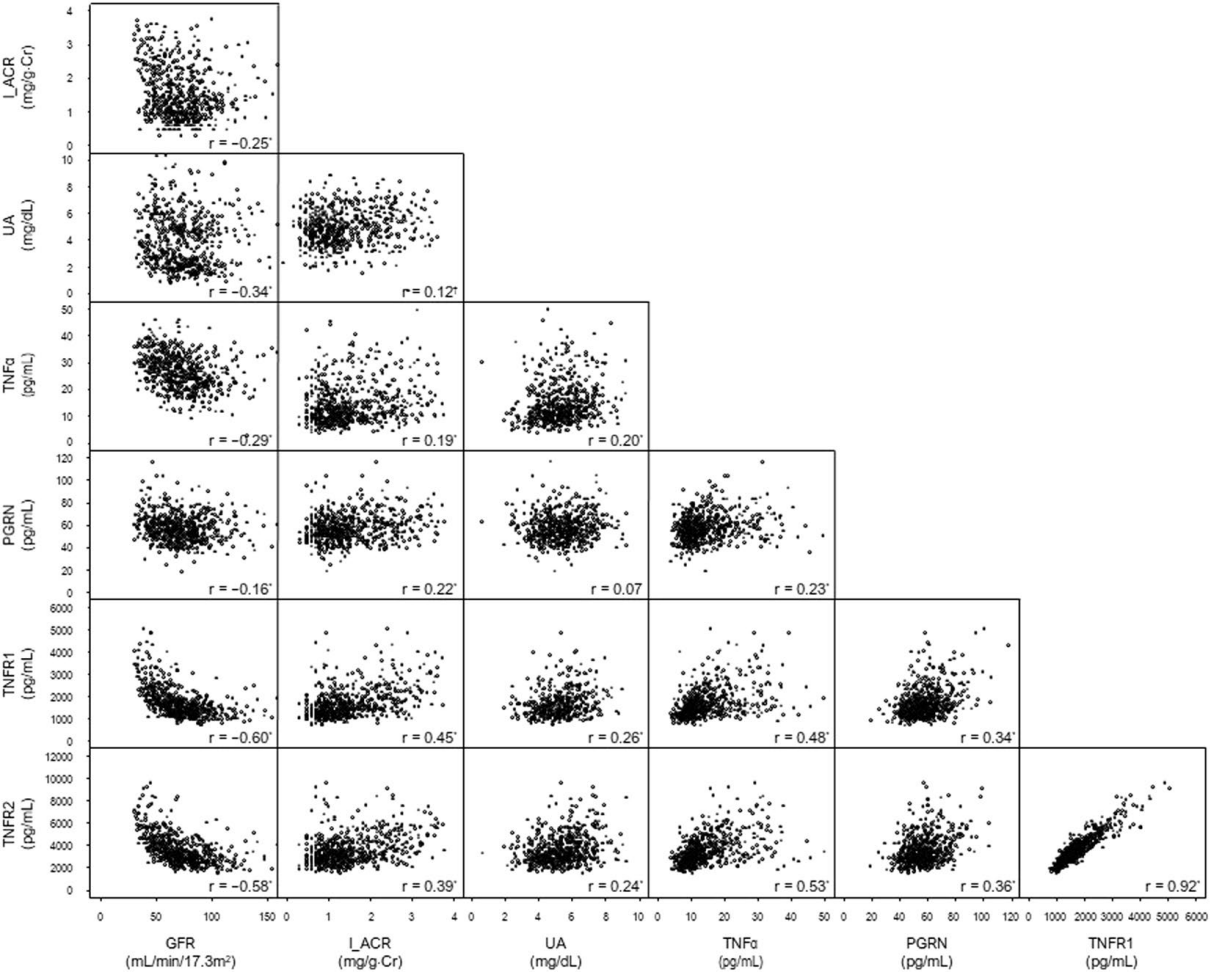
Pearson’s correlation coefficients of eGFR, common logarithmic transformed ACR (l_ACR), UA, and TNF-related biomarkers and their scatter plots * <0.0001, ^†^ <0.01.

As shown in Fig. 1, significant positive correlations among TNF-related biomarkers were observed with r-values ranging from 0.23 (TNFα vs. PGRN) to 0.92 (TNFR1 vs. TNFR2). Correlation between TNFα and its receptors (TNFRs) was stronger compared with that between PGRN and the receptors (TNFα–TNFR1, r = 0.48; TNFα–TNFR2, r = 0.53; PGRN–TNFR1, r = 0.34; PGRN–TNFR2, r = 0.36). All TNF-related biomarkers were also positively correlated with ACR with r-values ranging from 0.19 (TNFα) to 0.45 (TNFR1), and negatively correlated with eGFR with r-values ranging from −0.16 (PGRN) to −0.60 (TNFR1). UA is also associated with two renal traits [ACR (r = 0.12); eGFR (r = −0.34)] and TNF-related biomarkers other than PGRN [TNFα (r = −0.20); PGRN (r = 0.07); TNFR1 (r = −0.45); TNFR2 (r = −0.39)].

### TNF-related biomarkers and UA as risk factors of lower eGFR and increased albuminuria

All TNF-related biomarkers and many clinical covariates, such as age, sex, hemoglobin, HDL-C, HbA1c, ACR, and UA, were associated with lower eGFR (eGFR <60 mL/min/1.73 m^2^) in the univariate logistic regression analysis (Table 2). The prediction accuracy of each biomarker for risk of lower eGFR was low to moderate (UA, AUC 0.709; TNFα, AUC 0.703; PGRN, AUC 0.621; TNFR1, AUC 0.846; TNFR2, AUC 0.833) (Supplementary Table 2). Next, we calculated AUC to examine the additive benefit of TNF-related biomarkers or UA compared with covariates alone comprising age, sex, Sys BP, hemoglobin, HbA1c, and ACR (basic model) as risk factors of lower eGFR. Each TNF-related biomarker or UA was independently associated with lower eGFR even after adjustment for the basic model. However, TNFR ligands had no impact on eGFR when either TNFR or UA was included in the basic model (Data not shown). In addition, the AUC increased with inclusion of any of biomarkers other than PGRN: (UA, ΔAUC 0.049, p < 0.001; TNFα, ΔAUC 0.022, p = 0.007; PGRN, ΔAUC 0.013, p = 0.06; TNFR1, ΔAUC 0.064, p < 0.001; TNFR2, ΔAUC 0.052, p < 0.001). When either TNFR and UA were included in the basic model, the AUC further increased (Table 3).

**Table 2:** ORs for the risk factors of lower eGFR in study patients using clinical predictors and TNF-related biomarkers.

	OR^a^ (95% CI)	*P*
Age	1.08 (1.06–1.10)	<0.001
Sex	1.54 (1.08–2.19)	<0.001
Hemoglobin	0.70 (0.63–0.78)	<0.001
Sys BP	1.01 (0.998–1.02)	0.13
HbA1c	0.85 (0.73–0.99)	<0.001
HDL-C	0.98 (0.97–0.995)	0.008
ACR	1.85 (1.55–2.22)	<0.001
UA	1.81 (1.55–2.10)	<0.001
TNFα	1.97 (1.64–2.38)	<0.001
PGRN	1.53 (1.29–1.84)	<0.001
TNFR1	5.11 (3.86–6.78)	<0.001
TNFR2	4.79 (3.64–6.32)	<0.001

Abbreviations used in this table are the same as those in Table 1. OR, odds ratio; CI, confidence interval. ^a^ORs are per 1 increase or per 1-SD (ACR = 0.76; TNFα = 0.21; TNFR1 = 0.15; TNFR2 = 0.15; PGRN = 0.09) increase of each logarithm-transformed TNF-related biomarker.

**Table 3:** ORs and AUC for the risk factors of lower eGFR in study patients using clinical predictors, UA, and TNF-related biomarkers.Please see the attachment table 3. I would like to change the table frame.

	OR^a^ (95% CI)	*P*	AUC	Difference in AUC (95% CI)	*P*
Basic model (age, sex, hemoglobin, Sys BP, HbA1c, and ACR)	Ref.		0.812	Ref.	
Basic model + UA	2.00 (1.66–2.41)	<0.001	0.861	0.049 (0.026, 0.072)	<0.001
Basic model + TNFα	1.72 (1.39–2.13)	<0.001	0.834	0.022 (0.006, 0.038)	0.007
Basic model + PGRN	1.50 (1.21–1.85)	<0.001	0.825	0.013 (−0.0004, 0.026)	0.06
Basic model + TNFR1	4.03 (2.90–5.58)	<0.001	0.876	0.064 (0.039, 0.089)	<0.001
Basic model + TNFR2	3.29 (2.43–4.45)	<0.001	0.863	0.052 (0.028, 0.076)	<0.001
Basic model +UA +TNFR1	1.82 (1.49–2.23) 3.66 (2.60–5.16)	<0.001 <0.001	0.897	0.085 (0.057, 0.114)	<0.001
Basic model +UA +TNFR2	1.81 (1.49–2.20) 2.92 (2.06–4.11)	<0.001 <0.001	0.887	0.075 (0.048, 0.103)	<0.001

Abbreviations used in this table are the same as those in Tables 1 and 2. AUC, areas under the ROC; Ref, reference; ROC, receiver operating characteristic.

^a^ORs are per 1 (UA) increase or 1-SD (TNFa = 0.21; TNFR1 = 0.15; TNFR2 = 0.15; PGRN = 0.09) increase of each logarithm transformed TNF-related biomarker.

Next, we examined the additive benefit of TNF-related biomarkers or UA compared with the basic model alone as risk factors of increased ACR. In multivariate logistic regression analysis, eGFR was included as a covariate instead of ACR from the above basic model. Each TNF-related biomarker, but not UA, was independently associated with increased ACR (Micro) even after adjustment for the basic model. Moreover, PGRN showed an impact on ACR even if either TNFR was included in the basic model. Further, the inclusion of each TNF-related biomarker except TNFα in the basic model increased the AUC (PGRN, ΔAUC 0.029, p = 0.03; TNFR1, ΔAUC 0.066, p < 0.001; TNFR2, ΔAUC 0.048, p < 0.001) (Supplementary Table 3).

## Discussion

The major finding in this large cross-sectional study was that circulating TNF-related inflammatory biomarkers (TNFα, PGRN, TNFR1, and TNFR2) were associated with two important renal traits (ACR and eGFR) in Japanese patients with T2D and an eGFR ≥30 mL/min/1.73 m^2^. Among four TNF-related biomarkers, the association of both TNFRs (marginally stronger for TNFR1) with eGFR were the strongest after adjustment for relevant covariates. The performance of UA for the risk factor of lower eGFR appeared to be almost equivalent to each TNFR and superior to TNFR ligands. Note that each TNFR and UA were independently associated with renal traits after adjustment for relevant clinical covariates.

Serum UA levels are increased in patients with reduced eGFR, and those levels are associated with future eGFR decline^30^. This association is true even in patients with type 1 diabetes (T1D) and almost normal eGFR^31^. The same research group also demonstrated that those levels were an independent predictor of early GFR decline in a prospective cohort study of patients with T1D and normal eGFR^32^. A post hoc analysis of the Reduction of Endpoints in Non-insulin-dependent diabetes mellitus with the Angiotensin II Antagonist Losartan (RENAAL) trial showed that approximately 15% of losartan’s renal protective effect is attributed to the decrease in serum UA levels^33^. To date, there have been few randomized controlled studies evaluating the effect of serum UA lowering therapy^34^. Liu *et al*.^35^ demonstrated that long-term serum UA-lowering therapy using allopurinol increased eGFR in patients with T2D even though the study had a randomized open parallel-controlled design. Therefore, we have high expectations for the allopurinol study on preventing early renal function loss (PERL) in 400 patients with T1D and increased ACR^36^. In the present study, the impact of UA for risk of lower eGFR was equivalent to that of TNFRs but superior to that of TNFR ligands.

Krolewski *et al*.^5,6,13,14^ found that circulating levels of TNFRs were robust predictors of GFR decline in patients with both types of diabetes at various stages. Since then, studies from several groups have corroborated the usefulness of these biomarkers^7–9,37^. Elevated levels of TNFRs were also inversely associated with the percentage of endothelial cell fenestration and positively associated with the mesangial fractional volume in American Indians with T2D and preserved renal function, a population at very high risk of renal function decline^38^. Remarkably, the median levels of TNFRs in Pima Indians (TNFR1, 2833 pg/mL; TNFR2, 4835 pg/mL) were almost twice as high as those in the Joslin Kidney Study cohort, which was predominantly composed of Caucasians (TNFR1, 1310 pg/mL; TNFR2, 2527 pg/mL). In the present study, the median levels of TNFR1 and TNFR2 in patients with eGFR ≥60 mL/min/1.73 m^2^ were 1384 pg/mL and 2972 pg/mL, respectively. In addition, the cutoff values of TNFR1 and TNFR2 for risk of lower GFR (eGFR <60 mL/min/1.73 m^2^) were 1776 pg/mL and 3610 pg/mL, respectively, according to the ROC analysis. The levels of TNFRs in Japanese individuals appeared lower than those in Pima Indians, considering that approximately 90% of Pima Indian study participants had normal GFR (GFR ≥60 mL/min/1.73 m^2^). Thus, levels of TNFR in the Japanese population appear to be equivalent to those in Caucasians. The differences in the distribution of TNFR levels may be partly derived from the high degree of obesity. Further studies are required to determine whether TNFR levels differ among race.

PGRN was initially considered a pro-inflammatory adipokine induced by TNFα, but it has been considered to also have anti-inflammatory functions since Tang *et al*. showed that PGRN is a ligand for TNFRs and inhibits the TNF–TNFR signaling pathway^18,39^. Therefore, we measured PGRN in addition to TNFα in the present study although further studies are needed to clarify the precise basic mechanisms of PGRN underlying the pathogenesis of DKD. Richter *et al*.^21^ demonstrated that CKD stage (eGFR) was the strongest independent predictor of PGRN in 532 patients with stage 1–5 CKD, although they did not measure another important renal trait, ACR. In contrast, a more recent study showed that only ACR and serum Cr were associated with PGRN after adjustment for clinical covariates and the inflammatory markers interleukin-6 and TNFα in patients with T2D^22^. In our study, we partially confirmed their results of a stronger association between ACR and PGRN, compared with that between eGFR and PGRN. Of note, in the present study, both ligands of the TNFRs had no association with lower eGFR (eGFR <60 mL/min/1.73 m^2^) when either TNFR was included in the model as a covariate. However, PGRN but not TNFα was weakly associated with increased ACR (Micro) even after adjustment for either TNFR, suggesting that measurement of PGRN may be related to development of early renal injury in patients with diabetes.

It is unclear why circulating levels of TNFRs were more closely associated with eGFR than those of their ligands, because laboratory data regarding the role of TNF–TNFR and PGRN–TNFR pathways in the kidney are limited. A possible explanation is that because TNFR levels are at least 100 times higher than TNFα levels despite a certain level of correlation between TNFα and its receptors, circulating TNFRs may play a role in progression of DKD independent of TNFα levels, apart from functioning as decoys for TNFα. On the other hand, PGRN might have a low specific affinity for TNFRs compared to that of TNFα, based on the observation that TNFα–TNFRs correlation was stronger than PGRN–TNFRs correlation despite circulating levels of PGRN being at least 1000 times higher than those of TNFα.

It is possible that the correlation between TNF-related biomarkers and eGFR merely reflects reduced renal filtration by the kidney. In fact, we previously reported that the levels of TNFRs in hemodialysis patients are approximately 10-fold higher than those in the patients of the present study^40^. Further, the levels of all TNF-related biomarkers measured in the present study increased overall with reduced eGFR. However, this association may not be applicable for all patients, as shown in Fig. 1. In an isotope-labeled experiment, Bemelmans *et al*.^41^ showed that the levels of TNFα and TNFRs increased in bilateral nephrectomized mice and that the liver and lungs as well as the kidneys were involved in the clearance of TNFR. These findings suggest that the clearance of these molecules is closely related; however, it cannot explain all of our findings.

The main limitation of our study was its cross-sectional design. Therefore, our study did not establish a causal or temporal relationship among TNF-related biomarkers, UA, and renal traits. However, it should be noted that we recruited a substantial number of homogeneous Japanese patients with T2D and simultaneously measured two different ligands of TNFRs in addition to TNFRs. In conclusion, the present study provides evidence that TNFR ligands were associated with lower eGFR, but the associations were not as strong as those with TNFRs and UA in Japanese patients with T2D and an eGFR ≥30 mL/min/1.73 m^2^. These results also suggest that development of novel therapies targeted at inhibiting TNFRs as well as UA may be beneficial in prevention of DKD progression. The precise mechanism underlying the TNF–TNFR pathway in patients with diabetes requires further investigation.

## Electronic supplementary material

Supplementary Table 1–3
