# Supplementary material for: Association between circulating tumor necrosis factor-related biomarkers and estimated glomerular filtration rate in type 2 diabetes
Source: Sci Rep. 2018 Oct 17;8:15302. doi: 10.1038/s41598-018-33590-w (PMC6193030; doi:10.1038/s41598-018-33590-w)
Supplement: Supplementary file 1 — Supplementary Table 1–3 [file 41598_2018_33590_MOESM1_ESM.docx]

**Association between circulating tumor necrosis factor-related biomarkers and estimated glomerular filtration rate in type 2 diabetes**

Nozomu Kamei, MD, PhD^1,2,3$^, Mami Yamashita, MD^2,4$^, Yuji Nishizaki, MD, MPH, PhD^5^, Naotake Yanagisawa, PhD^5^, Shuko Nojiri, PhD^5^, Kanako Tanaka^2^, Yoshinori Yamashita, MD, PhD^3^, Terumi Shibata^6^, Maki Murakoshi, MD, PhD^6^, Yusuke Suzuki, MD, PhD^6^_,_ Tomohito Gohda, MD, PhD^6^

^1^Department of Endocrinology and Metabolism, Hiroshima Red Cross Hospital and Atomic-bomb Survivors Hospital, 1-9-6, Senda-machi, Naka-ku, Hiroshima 730-8619, Japan

^2^Department of Endocrinology and Diabetology, National Hospital Organization, Kure Medical Center and Chugoku Cancer Center, 3-1, Aoyama-cho, Kure-city, Hiroshima 737-0023, Japan

^3^Institute for Clinical Research, National Hospital Organization, Kure Medical Center and Chugoku Cancer Center, 3-1, Aoyama-cho, Kure-city, Hiroshima 737-0023, Japan

^4^Department of Molecular and Internal Medicine, Graduate School of Biomedical and Health Sciences, Hiroshima University, 1-2-3, Kasumi-cho, Minami-ku, Hiroshima 734-8551, Japan

^5^Juntendo University, Medical Technology Innovation Center, 2-1-1, Hongo, Bunkyo-ku, Tokyo 113-8421, Japan

^6^Department of Nephrology, Juntendo University Faculty of Medicine, 2-1-1, Hongo, Bunkyo-ku, Tokyo 113-8421, Japan

^$^Both authors contributed equally.

| Supplementary Table 1. Characteristics of the study group based on albuminuria level. | | | | |
| --- | --- | --- | --- | --- |
|  | Normoalbuminuria | Microalbuminuria | Macroalbuminuria |  |
| Characteristic | ACR < 30 | ACR 30–299 | ACR ≥ 300 | *P** |
|  | (n = 334) | (n = 171) | (n = 89) |  |
| ACR (mg/g·Cr) | 10 (6, 17) | 79 (40, 152) | 690 (449, 1487) |  |
| eGFR (mL/min/1.73 m^2^) | 72 (61, 86) | 69 (53, 85) | 55 (44, 70) | <0.001 |
| Age (yr) | 64 ± 13 | 66 ± 12 | 65 ± 12 | 0.48 |
| Male sex (%) | 52.4 | 57.9 | 61.8 | 0.07 |
| BMI (kg/m^2^) | 24.7 ± 4.4 | 25.3 ± 4.6 | 25.9 ± 5.0 | 0.10 |
| Sys BP (mmHg) | 137 ± 15 | 141 ± 18 | 145 ± 22 | <0.001 |
| UA (mg/dL) | 5.2 ± 1.3 | 5.5 ± 1.4 | 5.7 ± 1.4 | <0.001 |
| HDL-C (mg/dL) | 53 ± 13 | 51 ± 13 | 52 ± 13 | 0.18 |
| Non-HDL-C (mg/dL) | 129 ± 31 | 129 ± 34 | 132 ± 34 | 0.77 |
| Hemoglobin (g/dL) | 13.7 ± 1.8 | 13.6 ± 1.7 | 13.3 ± 1.7 | 0.20 |
| HbA1c (%) | 7.2 ± 1.1 | 7.5 ± 1.2 | 7.5 ± 1.3 | 0.005 |
| CRP (mg/dL) | 0.09 (0.06, 0.17) | 0.11 (0.06, 0.24) | 0.12 (0.07, 0.21) | 0.02 |
| TNF-related biomarkers | |  |  |  |
| TNFα (pg/mL) | 11.5 (9.0, 16.7) | 13.6 (9.8, 19.4) | 14.9 (11.2, 20.1) | <0.001 |
| PGRN (ng/mL) | 54 (48, 61) | 59 (50, 68) | 61 (54, 69) | <0.001 |
| TNFR1 (pg/mL) | 1401 (1175, 1725) | 1630 (1418, 2092) | 2229 (1751, 2811) | <0.001 |
| TNFR2 (pg/mL) | 3036 (2558, 3828) | 3522 (2841, 4570) | 4370 (3610, 5645) | <0.001 |
| *P value from unadjusted linear trend test. Data are mean ± SD, median (quartiles), or %. ACR, the ratio of urinary albumin to creatinine; BMI, body mass index; CRP, C-reactive protein; eGFR, estimated glomerular filtration rate; HbA1c, hemoglobin A1c; HDL-C, high-density lipoprotein cholesterol; PGRN, progranulin; SD, standard deviation; Sys BP, systolic blood pressure; TNFR, TNF receptor; UA, uric acid | | | | |

| Supplementary Table 2. Cutoff value and AUC of each biomarker for the risk of lower eGFR. | | | | | |
| --- | --- | --- | --- | --- | --- |
| Variable | Sensitivity (%) | Specificity (%) | Distance | Cutoff value | AUC |
| UA (mg/dL) | 68.2 | 64.7 | 0.475 | 5.5 | 0.709 |
| TNFα (pg/mL) | 73.4 | 59.5 | 0.485 | 12.5 | 0.703 |
| PGRN (ng/mL) | 56.3 | 60.9 | 0.586 | 58 | 0.621 |
| TNFR1 (pg/mL) | 70.8 | 81.6 | 0.345 | 1776 | 0.846 |
| TNFR2 (pg/mL) | 78.1 | 74.4 | 0.337 | 3610 | 0.833 |
| Abbreviations used in this table are the same as those in Supplementary Table 1. AUC, area under the ROC; ROC, receiver operating characteristic | | | | | |

| Supplementary Table 3. ORs and AUC for the risk factors of microalbuminuria in study patients using clinical predictors and TNF-related biomarkers. | | | | | |
| --- | --- | --- | --- | --- | --- |
|  | OR^a^ (95% CI) | *P* | AUC | Difference in AUC (95% CI) | *P* |
| Basic model (age, sex, hemoglobin, Sys BP, HbA1c, and eGFR) | Ref. |  | 0.662 | Ref. |  |
| Basic model + UA | 1.14 (0.99–1.32) | 0.07 | 0.667 | 0.004 (−0.007, 0.016) | 0.46 |
| Basic model + TNFα | 1.34 (1.11–1.60) | 0.002 | 0.679 | 0.017 (−0.003, 0.036) | 0.09 |
| Basic model + PGRN | 1.50 (1.25–1.79) | <0.001 | 0.691 | 0.029 (0.004, 0.055) | 0.03 |
| Basic model + TNFR1 | 2.28 (1.77–2.93) | <0.001 | 0.728 | 0.066 (0.031, 0.100) | <0.001 |
| Basic model + TNFR2 | 1.96 (1.54–2.50) | <0.001 | 0.710 | 0.048 (0.016, 0.079) | 0.003 |
| Basic model + |  |  |  |  |  |
| TNFR1 | 2.08 (1.60–2.70) | <0.001 | 0.736 | 0.074 (0.038, 0.109) | <0.001 |
| PGRN | 1.30 (1.07–1.59) | 0.007 |  |  |  |
| Basic model + |  |  |  |  |  |
| TNFR2 | 1.76 (1.36–2.27) | <0.001 | 0.718 | 0.056 (0.023, 0.089) | <0.001 |
| PGRN | 1.31 (1.09–1.60) | 0.005 |  |  |  |
| Abbreviations used in this table are the same as those in Supplementary Table 1 and Supplementary Table 2. CI, confidence interval; OR, odds ratio; Ref, reference ^a^ORs are per 1 (UA) increase or 1-SD (TNFα = 0.21; TNFR1 = 0.15; TNFR2 = 0.15; PGRN = 0.09) increase of each logarithm transformed TNF-related biomarker. | | | | | |
